# Mineral and Anti-Nutritional Contents of Niger Seed (*Guizotia abyssinica* (L.f.) Cass., Linseed (*Linumusitatissimum* L.) and Sesame (*Sesamumindicum* L.) Varieties Grown in Ethiopia

**DOI:** 10.3390/foods6040027

**Published:** 2017-04-01

**Authors:** Tesfaye Deme, Gulelat D. Haki, Nigussie Retta, Ashagrie Woldegiorgis, Mulatu Geleta

**Affiliations:** 1 Center for Food Science and Nutrition, College of Natural Sciences, Addis Ababa University, Box 1176, Addis Ababa, Ethiopia; negussie.retta@gmail.com (N.R.); ashuyz1@yahoo.com (A.W.); 2 Department of Food Science and Nutrition, Jigjiga University, Box 1020, Jigjiga, Ethiopia; 3 Department of Food Science and Technology, Botswana University of Agriculture and Natural Resources, Private Bag 0027, Gaborone, Botswana; gulelatw@yahoo.com; 4 Department of Plant Breeding, Swedish University of Agricultural Sciences, Box 101, SE-23053 Alnarp, Sweden; mulatu.geleta.dida@slu.se

**Keywords:** Nigerseed, Linseed, Sesame, mineral, phytate, tannin

## Abstract

Oilseeds are rich sources of micronutrients and contribute to combating malnutrition caused by micronutrient deficiency. The objective of this study was to investigate the mineral and anti-nutritional contents of different varieties of niger seed, linseed and sesame. Five niger seed, eight linseed and ten sesame varieties were used. Inductively Coupled Plasma Atomic Emission Spectrometry (ICP-AES) was used for mineral analysis and the standard method was adopted to estimate tannin and phytate. Twelve mineral elements; Ca, K, Mg, Na, P, B, Cu, Fe, Mn, S, Se and Zn were analyzed for each oilseed variety. In niger seed, phosphorous was the most abundant mineral element ranging from 661 to 867 mg/100 g and selenium was the least, ranging from 0.1 to 0.33 mg/100 g. Potassium was recorded in the range of 502 to 732 mg/100 g for linseed varieties. Calcium was the most common mineral element in sesame (1112 to 1787 mg/100 g). The average phytate contents of niger seed, linseed and sesame varieties were353 mg/100 g, 104 mg/100 g and 285 mg/100 g, respectively. Tannin ranged from 91 to 201 mg/100 g, 96 to 695 mg/100 g and 85 to 660 mg/100 g in niger seed, linseed and sesame, respectively. In conclusion, there is a significant variation among the varieties within each crop species as well as among the different oilseeds in terms of their mineral and anti-nutritional contents.

## 1. Introduction

Niger seed (*Guizotia abyssinica* (L.f.) Cass.), Linseed (*Linumusitatissimum* L.) and Sesame (*Sesamumindicum* L.) are major oil seed crops grown in Ethiopia [1]. Oilseeds are leading suppliers of superior quality and specialty vegetable oils to nutritional products, natural food and premium snack foods worldwide [2]. Oilseeds are rich sources of nutrients and also have wide range of medicinal values. They add important nutritional values to diets as they are sources of vegetable oil, high quality proteins as well as oil soluble vitamins like vitamin A. Though there are oil-bearing tree fruits which come first in terms of oil content, oilseeds are the largest source of vegetable oils [3].

Oilseeds are primarily used for the extraction of edible oils. The oilseed cake or the meal obtained after extraction of oil is largely used as cattle feed or fertilizer. The defatted meal is a rich source of protein and, if suitably processed, has a great potential as food for human consumption. Some oilseeds are consumed as such and, though in small amounts, contribute to the daily intake of several nutrients [4]. The dietary requirement of minerals is relatively small which is in the range of 1–2500 mg per day depending on the type of mineral [5]. Despite the fact that minerals are required in a relatively small quantity, they play a vital role in forming blood and bone, transmitting impulses from our nervous system to our extremities and vice versa and generally maintaining optimal health. Minerals may be broadly classified as macro (major) or micro (trace) elements. The macro-minerals, also called macronutrients, include calcium, phosphorus, sodium, chloride, potassium and magnesium and are needed in amounts of >50 mg/day. The micro-minerals, also called micronutrients, include iron, copper, cobalt, iodine, zinc, manganese, molybdenum, fluoride, chromium and selenium and are needed in amounts of <50 mg/day. Minerals known as ultra-trace elements such as Aluminum, Arsenics, Barium, Boron and Bromine are required in extremely small quantities (in µg) per day [6].

Inadequate intake of micro nutrients is a major public health problem, especially in populations of developing countries in which pregnant women and children are affected [6]. Since infants need proper amount and type of micronutrients for their healthy body growth, they need special attention in supplementing with micronutrients [7]. Different oilseeds like niger seed, linseed and sesame can be used as sources of micronutrients. To assess the dietary intake and adequacy of minerals, information needs to be collected on mineral element content of foods, diets and water [8,9]. In view of the importance of macro and micro mineral elements in the nutrition of humans and animals and their metabolic inter-relationships which influences other vital factors needed for the survival of living organisms like enzymes, anti-oxidants, vitamins, etc., it is important to regularly obtain up-to-date information on the minerals content of the variously commonly consumed plant foods used for human and animal foods and feeds respectively e.g. legumes, cereals, fruits and vegetables. This is because of the effects of chemicals like pesticides, herbicides, fertilizers on the mineral contents of the soils where these plants are cultivated. Genetic, location and environmental factors could also influence the levels of the mineral elements in plants [5].

Oil seeds were studied for their micronutrient content with the objective of determining their composition. The mineral composition of Saudi Arabian white and dark sesame cultivars was reported by Alyemeni et al. [10] using an Atomic Absorption Spectrophotometry (AAS) procedure. The analysis revealed the following amount for white and dark sesame cultivars: Na (78 and 72), K (382 and 374), Ca (1228 and 1200), Mg (178 and 185), Fe (10.4 and 10.6), Zn (3.6 and 3.8) and P (598 and 580) mg/100 g. The mineral composition of niger seed collected from different regions of Ethiopia and studied using flame atomic absorption spectrometry was also reported: Na (159–736), K (5594–8203), Ca (340–680), Mg (2404–4656), Mn (13.4–34.3), Fe (31.6–370), Cu (9.5–61.2), Zn (23.4–46.2) µg/g [11]. Linseed was also investigated for its mineral composition using AAS [12] and the analysis revealed the following: K (822.1), Ca (236.4), Mg (422.5), Na (30.12), Fe (6.01), Zn (4.43), Mn (2.73) and Cu (1.9) mg/100 g.

The availability of macro and micro-nutrients to our body is affected by anti-nutritional factors found in foods from plant sources. Anti-nutritional factors form complexes with nutrients and hence block micronutrients to be absorbed by body cells. Oil seeds contain significant amount of phytic acid, fibers and other binding agents that can reduce bioavailability of minerals obtained from the seeds. Phytic acid, the hexaphosphate of myoinositol, functions as the chief storage form of phosphate and inositol in mature seeds. Whole oilseeds contain about 1.5%, while some oilseed protein concentrates can contain over 7.0% of phytic acidon a dry seed basis. Phytic acid is a strong chelating agent that can bind to mono- and di- valent metal ions to form complex phytate. Research reports from different animal feeding trials suggest poor bio-availability of minerals such as zinc, calcium, magnesium, phosphorus and possibly iron from diets containing high phytate foods [13].

Plant breeding in Ethiopia for the improvement of nutritional qualities of crops is highly limited. Consequently, little is known about the macro and micro mineral contents of different crop varieties that the agricultural research centers have released to farmers. Hence, this study aimed at determining the mineral composition and anti-nutritional factors of different varieties of niger, linseed and sesame in order to contribute to the identification of suitable varieties that can be used in the breeding programs and thereby promote the livelihood of farmers and consumers.

## 2. Material and Methods

### 2.1. Sample Preparation

Seed samples were obtained from two research centers of the Ethiopian Institute of Agricultural Research (EIAR). Five varieties of niger seed (*Shambu*, *Kuyu*, *Esete*, *Ginchi* and *Fogera*) and eight varieties of linseed (*Chilalo*, *Tole*, *Ci-1652*, *Kassa-2*, *Bekoji*, *Berene*, *Ci-1525* and *Belaye-96*) were obtained from Holeta Agricultural Research Center and ten varieties of sesame (*K-74*, *S*, *M-80*, *E*, *Serkamo*, *Tate*, *Argene*, *T-85*, *Adi* and *Abasena*) were obtained from Melaka Werer Agricultural Research Center. The growing conditions (soil type, duration of cultivation, rain fall, altitude) for niger seed and linseed varieties are similar, i.e., all varieties of both crops were grown in the same area (altitude 2400 m above sea level, temperature range 6 °C to 22 °C, rain fall 1144 mm, and soil type vertisol). The same is true of sesame seed varieties, i.e., they were grown in the same area with the same environmental conditions (altitude 750 m, temperature26.7 °C to 40.8 °C, rain fall 590 mm, and soil type vertisol). Variation in seed yield and oil content are among major factors that differentiate different varieties of each crop used in the present study. All impurities including broken seeds were manually cleaned out from each seed sample before they were used for analyses.

### 2.2. Mineral Content Analysis

Seed samples of the three oil crops were ground to a fine powder with a Kinematica A10 Grinder (Kinematica, Lucerne, Switzerland). Five hundred milligrams (0.5 g) of flour of each sample was packed in a Teflon capsule and placed in a microwave oven (Microwave Accelerated Reaction System 5, CEM Corporation, Matthews, NC, USA, with a regulated pressure and temperature). Each sample was digested in 10 mL solution (7 mL of concentrated nitric acid and 3 mL of water) which was then diluted with water up to 50 mL before analysis. The mineral content analysis of the samples was conducted at the Instrumental Chemistry Laboratory, Department of Biology, Lund University, Sweden, using Inductively Coupled Plasma Atomic Emission Spectrometry (ICP-AES, OPTIMA 8300, Perkin-Elmer, Waltham, MA, USA). Perkin-Elmer, Special purpose examination (SPEX), AccuStandard and Merck atomic spectrometry standards, Germany, were used for this analysis. The mineral content was recorded in milligram of mineral per 100 g of flour (mg/100 g).

### 2.3. Anti-Nutrient Content Analyses

#### 2.3.1. Phytate

The phytate content of the samples was determined according to the method described in Oyaizu [14]. One hundred milligram of sample was extracted with 10 mL of 2.4% HCl in a mechanical shaker for one hour at a room temperature. The extract was centrifuged at 3000 rpm for 30 min. The clear supernatant was used for phytate estimation. One milliliter of Wade reagent (containing 0.03% solution of FeCl_3_·6H_2_O and 0.3% of sulfosalicylic acid in water) was added to 3 mL of the sample solution (supernatant) and the mixture was vortexed for 5 s. Absorption readings at 500 nm were taken against a blank solution (3 mL extract solution mixed with 2 mL of 2.4% HCl). Sodium salt of phytic acid (4.5–36 mg/mL) was used as standard for construction of calibration curve.

#### 2.3.2. Condensed Tannin

Tannin was determined using Burns [15] method modified by Maxson and Rooney [16]. One gram of each sample was weighed and mixed with 10 mL of 1% HCl in methanol in a screw cap test tube. Then, the tube was shaken for 24 h at room temperature on a mechanical shaker. The solution was centrifuged at 1000 rpm for 5 min. One mL of supernatant was transferred to another test tube and mixed with 5 mL of vanillin-HCl reagent (prepared by mixing equal volume of 8% concentrated HCl in methanol and 4% vanillin in methanol). A mixture of 1 mL of extract solution with 5 mL of 1% HCl without vanillin-HCl reagent was used as a blank. After 20 min, the absorbance of the solutions and the standard were measured using Shimadzu UV-1800 at 500 nm. (+) catechin (0.5–12 mg/100 mL) was used as standard for construction of calibration curve.

### 2.4. Statistical Analysis

This study was designed to evaluate the mineral and anti-nutritional content of selected edible oilseeds (niger, linseed and sesame) grown in Ethiopia. The results were expressed as mean values and standard deviation (SD) of duplicate measurements. The average values of each oil seeds were also compared. The results were analyzed using one-way analysis of variance (ANOVA) followed by Duncan’s test for mean separation with α ˂0.05. SPSS version 20.0 package (SPSS, IBM corp., Chicago, IL, USA) was used for analysis.

## 3. Results and Discussion

Table 1 presents the content of macro and micro minerals and anti-nutrients in niger seed varieties. There is a significant variation within the varieties. Phosphorous (P) constitutes the highest amount among the macro-minerals ranging from 660.61 mg/100 g (*Esete*) to 867.02 mg/100 g (*Kuyu*) followed by potassium (K) with values in the range of 610.15 mg/100 g (*Esete*) to 808.65 mg/100 g (*Ginchi*). Other macro-minerals (Ca, Mg, S and Na) also showed significant variation within the varieties to the extent that allows selecting varieties for further breeding to improve specific group of minerals. This study clearly indicates that niger seed is a good source for these minerals. The average amount of calcium recorded in the present study was lower than that reported in Rao [8]. The difference in soil types where the plants were grown and the genetic variation among the varieties are among the most likely reasons for the differences reported in the two studies. In addition, the method and the instrument sensitivity are possible sources of the difference.

The anti-nutritional factors, tannin and phytate, of niger seed show significant variation among the varieties. The average phytate content in this study was 352.58 mg/100 g and higher than what was reported by Baranwal and Bhatnagar [17]. The average tannin content in niger seed was 134.6 mg/100 g which slightly differ from Rao [8] who reported 158 mg/100 g but higher than what was reported in Baranwal and Bhatnagar [17]. The levels of these substances in plants vary with the specie of plant, cultivar and post-harvest treatment [4].

The micro-minerals analyzed in this study varied from 5.65 mg/100 g (*Esete*) to13.65 mg/100 g (*Kuyu*) for Fe, followed by Zn with values in the range of 3.96 mg/100 g (*Esete*) and 3.57 mg/100 g (*Ginchi*). Overall, the average values of micro-minerals were in the range of 0.182 mg/100 g (Se) and 10.26 mg/100 g (Fe). The finding from this study is a good indicator that it is possible to develop niger seed varieties with good micronutrient contents.

The macro- and micro-minerals and anti-nutritional content for linseed varieties are given in Table 2. The analysis revealed that there is significant variation among the varieties in both macro- and micro-mineral contents. Potassium is the principal macro-mineral with the value ranging from 501.51 mg/100 g (for *Tole*) to 732.37 mg/100 g (for *Berene*) followed by phosphorous, magnesium, calcium, sulfur and sodium, respectively. Micro-minerals analyzed also showed significant variation among the varieties analyzed. Fe is the most common micro-mineral, and is followed by Mn, Zn, B, Cu and Se in that order.

The result obtained in this study is in line with the work reported by Kiralan et al. [18] except for the findings relating to sulfur and selenium, which the report didnot include. In the study reported by Kiralan et al. [18], linseed lines from four European countries that were grown under the same environmental conditions in replicates were included. The study concluded that the difference in mineral composition among lines is mainly due to the cultivars genetic makeup and the genotype environment interactions, as little variation due to environment is expected. Similarly, the different varieties of the three oil crops included in the present study were grown under the same environmental conditions before the progeny seeds were analyzed. Hence it is safe to conclude that genotypic variation is a major factor behind the significant variation in the contents of different minerals among the varieties. In addition to the micronutrients, anti-nutritional factors, phytate and tannin in different linseed varieties were analyzed, and significant differences among the varieties were found. As it is shown in Table 2, variety *Kassa-2* had the highest phytate content with mean value of 156.91 mg/100 g whereas variety *Berene* had the lowest with mean value of 47.06 mg/100 g. Unlike phytate, the highest and lowest tannin content were recorded in variety Bekoji (mean value = 695 mg/100 g) and variety *Kassa-2* (mean value = 96 mg/100 g), respectively. The variation in phytic acid contents of different varieties could be due to phosphorus utilization efficiency of the varieties [19], which is the result of the genetics behind this trait. This indicates the possibility of reducing the anti-nutritional contents of some of the varieties through cross-breeding. Reducing anti-nutritional contents in oil seeds is important, as it affects the availability of minerals. The average phytate content of Ethiopian linseed varieties revealed in the present study is lower than that reported in Oomah et al. [19] and Russo and Reggiani [20] for Canadian and Italian linseed, respectively. Oomah et al. [19] reported that phytic acid content is influenced by difference in location and year of production, variety (cultivar) and their interaction. Hence, the lower phytate content recorded in the present study as compared to these two previous studies can be attributed to the combinations of these factors.

The tannin content revealed in this study for Ethiopian linseed was higher than that reported for Italian linseed.

The mineral and anti-nutritional factors of different sesame varieties showed significant variation (Table 3), which can be partly attributed to genetic variation among the varieties [4]. Among the macro minerals analyzed, calcium comes first with an average value of 1463.45 mg/100g with variety *Tate* showing the highest calcium content (1786.5 mg/100 g); followed by phosphorous, potassium, magnesium, sulfur and sodium. The average values of these macro minerals were 743.59 mg/100 g, 506.69 mg/100 g, 364.82 mg/100 g, 322.17 mg/100 g, and 3.35 mg/100 g in that order. Similar to the case in linseed, the result obtained in this study differs to a certain extent from that reported in Deosthale [4] for sesame, which is the effect of genotypes, environments and the interactions between the two as well as the sensitivity of the instruments used for analysis.

Among the micro minerals iron is leading with an average value of 11.86 mg/100 g (with variety *Tate* showing the highest content: 22.63 mg/100 g) followed by zinc (5.36 mg/100 g), copper (1.73 mg/100 g), manganese (1.36 mg/100 g), boron (1.23 mg/100 g) and selenium (0.14 mg/100 g). The analysis of anti-nutritional content of sesame varieties showed that variety *Tate* had the highest phytate content (435.45 mg/100 g) whereas variety *Argene* had the lowest (224.92 mg/100 g). The tannin content of the sesame varieties ranged from 85 mg/100 g (in variety *E*) to 660 mg/100 g (in variety *Tate*). The average values obtained for potassium and magnesium as well as for iron and copper are in good agreement with the work reported by Elleuch et al. [21]. Whereas, the values obtained in this study for calcium and phosphorous are higher than that reported by Elleuch et al. [21] which can be attributed to combinations of environmental and genetic factors as well as the methods used for analysis. The phosphorous, zinc, manganese and copper contents obtained in this study are in a good agreement with the work reported by Özcan and Akbulut [22]. Similarly, the average calcium content in this study is consistent with the work reported by Gopalan et al. [23] for Indian cultivars. In contrast, the selenium content analyzed in this study is higher than the data reported by Makinde and Akinoso [24].

The results obtained for anti-nutritional contents (phytic acid) of sesame in the present study are lower than that reported by Phescatcha et al. [25] for black sesame except for variety *Tate*, which had comparable content. Makinde and Akinoso [24] found higher amount of phytic acid in larger seeds of white sesame (62.67 mg/100 g) than in smaller seeds of black sesame (52.60 mg/100 g), which are lower values when compared with results of the current study. Based on their results, Makinde and Akinoso [24] suggested that the amount of the phytic acid depends on seed size in sesame. Also, Lyon [26] reported that the phytic acid contents of the sesame varieties were affected by genotypes. The tannin content of the majority of the sesame varieties in this study is similar to that of brown and white sesame seed cultivars studied by Embaby [27]. The average value of tannin obtained in this study was within the range reported for brown and white sesame seeds (180–270 mg/100 g) by Embaby [27].

Comparison of the average values of mineral and anti-nutritional content of the three oilseed crops resulted in significant variation. The calcium content of sesame (1463.45 mg/100 g) is much higher than that of niger seed (424.66 mg/100 g) and linseed (208.68 mg/100 g) which indicates sesame as a best source of calcium among the major edible crops grown in Ethiopia. The average calcium content of different sesame varieties obtained in this study is similar with the results obtained by Gopalan et al. [23] for the Indian cultivars. The phosphorous content of niger seed (784 mg/100 g) and sesame seed (743.9 mg/100 g) was also higher than that of linseed (461.35 mg/100 g) and the variation in phosphorous content between niger and sesame seeds is statistically significant.

The average potassium and boron content of the three oil seeds is in the order of niger seed > linseed > sesame seed and the magnesium, sulfur and iron content is in the order of sesame > niger seed > linseed. The average level of selenium and manganese in the three oil seed crops is linseed > niger seed> sesame. The phytic acid content in niger seed varieties is higher than that of linseed and sesame varieties. On the other hand, the tannin content of linseed is higher than that of niger seed and sesame. The phytic acid content among the varieties of the three oil seed crops is significantly different. The phytic acid and mineral contents of oil crops were reported to vary with cultivar, location, year of production and their interaction [19,28].

## 4. Conclusions

Data presented here show that oilseeds are rich sources of both macro- and micro- minerals, and these oilseeds contain high proportions of fibrous hull and their phosphorus is mostly present in the form of phytate. Both components are known to limit the bioavailability of the metal ions [13]. The oilseed crops included in this study are rich in macro and micro minerals and can play a great role in combating malnutrition. The composition illustrates consumption of this products as such might contribute in replacing micronutrient supplementation like zinc and iron. Significant variation was observed among the varieties within each oil crop as well as among the different oil crops in their mineral and anti-nutrient content. Among sesame varieties, *Tate* showed high calcium, tannin and phytate content of 1786.5 mg/100 g, 660 mg/100 g, and 435 mg/100 g respectively. Variety *Berene* was the richest in potassium content among linseed varieties with composition of 732.37 mg/100 g. Phosphorous was the highest observed inorganic nutrient for *Kuyu* (Niger seed) with the value of 867.02 mg/100 g. Hence, breeders of these oil crops should make use of the results of the present study when selecting varieties for further breeding for improvement of target nutrients.

## Figures and Tables

**Table 1:** Minerals (macro and micro) and tannin and phytate contents of different niger varieties (in mg/100 g, wet base).

Minerals	Niger Seed Varieties					
	Shambu	Kuyu	Esete	Ginchi	Fogera	Average *
P	831.48^c^	867.02^a^	660.61^e^	703.63^d^	860.46^b^	784.64^a^
K	742.66^d^	767.4^c^	610.15^e^	808.65^a^	788.64^b^	743.50^a^
Ca	402.07^d^	450.72^b^	430.97^c^	371.94^e^	467.58^a^	424.66^b^
Mg	313.76^c^	352.95^a^	264.93^e^	290.82^d^	342.93^b^	313.08^b^
S	236.56^e^	266.35^b^	254.55^c^	276.67^a^	252.79^d^	257.38^b^
Na	1.65^d^	1.64^d^	2.46^c^	3.87^b^	4.97^a^	2.92^c^
Fe	9.46^c^	13.65^a^	5.65^d^	9.37^c^	13.17^b^	10.26^b^
Zn	3.94^a^	3.77^b^	3.96^a^	3.57^c^	3.74^b^	3.80^c^
Mn	1.83^d^	3.44^c^	1.05^e^	7.36^a^	3.63^b^	3.46^b^
B	1.96^a,b^	1.87^b,c^	2.1^a,b^	1.72^c^	2.23^a^	1.98^a^
Cu	0.93^a^	0.95^a^	1.04^a^	1.12^a^	1.07^a^	1.02^c^
Se	0.19^b^	0.17^b^	0.12^c^	0.33^a^	0.10^d^	0.18^b^
Tannin	109^c^ ± 7.12	167^b^ ± 1.57	91^d^ ± 3.46	105^c^ ± 8.41	201^a^ ± 2.62	134.6
Phytate	315.51^c^ ± 1.27	250.95^e^ ± 0.49	515.92^a^ ± 0.60	303.79^d^ ± 0.46	376.73^b^ ± 2.75	352.58

Values followed by different superscript in the same row are significantly different at *p* < 0.05. * Comparison is made with the average value of linseed and sesame seed.

**Table 2:** Minerals (macro and micro) and tannin and phytate content of different Linseed varieties (mg/100 g, wet base).

Minerals Analyzed	Linseed Varieties								
	Chilalo	Tole	Ci-1652	Kassa-2	Bekoji	Berene	Ci-1525	Belaye-96	Average *
K	643.52^b^	501.51^h^	518.13^g^	526.39^e^	531.5^d^	732.37^a^	573.67^c^	524.17^f^	568.91^b^
P	576.39^b^	352.68^h^	408.45^f^	442.42^d^	427.73^e^	579.48^a^	504.33^c^	399.31^g^	461.35^c^
Mg	338.53^a^	277.06^h^	315.04^d^	305.26^f^	303.93^g^	326.26^c^	335.14^b^	309.93^e^	313.89^b^
Ca	229.6^b^	173.6^g^	192.68^e^	211.28^c^	260.51^a^	200.63^d^	212.45^c^	188.72^f^	208.68^c^
S	195.72^b^	177.72^e^	189.34^c^	180.26^d^	170.23^f^	178.47^d,e^	199.94^a^	187.88^c^	184.95^c^
Na	13.14^d^	9.17^h^	9.56^g^	20.73^a^	13.35^c^	16.74^b^	12.28^e^	10.94^f^	13.24^a^
Fe	8.33^b^	7.47^d^	7.34^d^	10.29^a^	6.44^e^	7.75^c^	7.45^d^	7.3^d^	7.8^c^
Zn	4.04^c^	3.69^e^	4.15^b,c^	4.53^a^	4.13^bc^	4.63^a^	4.27^b^	3.87^d^	4.16^b^
Mn	5.36^a^	2.93^f^	4.2^c^	2.66^g^	3.8^d^	3.64^d^	3.35^e^	4.46^b^	3.8^a^
B	1.9^a^	1.59^b,c^	1.44^cd^	1.67^b^	1.6^b,c^	1.42^d^	1.51^b–d^	1.47^c,d^	1.58^b^
Cu	1.26^a^	.96^b^	1.24^a^	1.26^a^	1.26^a^	1.26^a^	1.35^a^	1.3^a^	1.24^b^
Se	0.21^b^	0.19^d^	0.1^g^	0.28^b^	0.18^e^	0.29^a^	0.22^c^	0.11^f^	0.2^a^
Tannin	168^e^ ± 5.64	161^e^ ± 3.35	199^c^ ± 6.57	96^f^ ± 1.84	695^a^ ± 6.43	185^d^ ± 1.88	204^c^ ± 4.1	309^b^ ± 3.78	252.13
Phytate	71.61^f^ ± 3.58	147.21^b^ ± 0.09	90.19^e^ ± 0.01	156.91^a^ ± 0.34	110.04^c^ ± 0.51	47.06^g^ ± 0.41	112.93^c^ ± 0.91	95.56^d^ ± 0.65	103.94

Values followed by different superscripts in the same row are significantly different at α<0.05. * Comparison is made with the average value of niger seed and sesame seed.

**Table 3:** Mineral (macro and micro) and tannin and phytate content of different Sesame seed varieties (mg/100 g, wet base).

Minerals Analyzed		Sesame seed varieties									
	Kelafo-74	S	M-80	E	Serkamo	Tate	Argene	T-85	Adi	Abasena	Average *
Ca	1490.38^f^	1559.54^b^	1366.69^g^	1520.37^e^	1557.74^b,c^	1786.5^a^	1526.64^d^	1556.2^c^	1111.61^i^	1158.83^h^	1463.45^a^
P	747.55^d^	741.73^e^	708.71^i^	800.58^a^	755.74^c^	736.36^f^	763.47^b^	722.34^h^	729.89^g^	729.48^g^	743.59^b^
K	515.12^c^	493.41^g^	476.64^h^	508.23^d^	535.73^a^	527.67^b^	502.57^e^	497.23^f^	493.23^g^	517.05^c^	506.69^c^
Mg	365.03^c^	372.33^b^	342.78^h^	401.35^a^	372.51^b^	355.79^f^	363.91^d^	362.6^e^	349.13^g^	362.79^e^	364.82^a^
S	304.48^h^	293.34^j^	320.56^f^	329.96^c^	326.5^d^	324.27^e^	311.67^g^	301.64^i^	341.92^b^	367.36^a^	322.17^a^
Na	4.5^c^	0.94^g^	2.21^f^	1.25^g^	5.83^b^	8.79^a^	3.14^e^	3.83^d^	.94^g^	2.05^f^	3.35^b^
Fe	14.77^d^	5.85^j^	7.66^g^	7.34^h^	18.07^b^	22.63^a^	15.37^c^	11.74^e^	6.8^i^	8.38^f^	11.86^a^
Zn	5.13^e,f^	5.23^e^	4.96^g^	6.25^a^	5.46^d^	5.87^b^	4.96^g^	4.98^g^	5.04f^g^	5.7^c^	5.36^a^
Cu	1.87^a^	1.63^c,d^	1.67^a–d^	1.73^a–d^	1.84^a,b^	1.66^b–d^	1.56^d^	1.73^a–d^	1.77^a–c^	1.87^a^	1.73^a^
Mn	1.44^ab^	1.37^bc^	1.23^c,d^	1.16^d^	1.45^a,b^	1.57^a^	1.45^a,b^	1.15^d^	1.45^a,b^	1.36b^c^	1.36^c^
B	1.25^b^	1.25^ab^	1.25^b^	1.2^ab^	1.15^b^	1.25^ab^	1.2^a,b^	1.15^b^	1.25^a,b^	1.35^a^	1.23^c^
Se	0.10^d^	0.18^b^	0.15^c^	0.19^b^	0.09^d,e^	ND	0.07^f^	0.15^c^	0.25^a^	0.08^e^	0.14^c^
Tannin	137^e^ ± 0.03	467^b^ ± 0.07	142^e^ ± 0.06	85^g^ ± 0.05	395^c^ ± 0.04	660^a^ ± 0.11	224^d^ ± 0.03	135^e^ ± 0.00	112^f^ ± 0.03	89^g^ ± 0.01	244.6
Phytate	292.75^c^ ± 0.3	233.81^g^ ± 0.04	275.76^e^ ± 0.7	230.81^h^ ± 1.1	316.79^b^ ± 0.9	435.45^a^ ± 0.5	224.92^i^ ± 0.1	291.46^d^ ± 0.3	267.71^f^ ± 0.3	275.65^e^ ± 0.2	284.511

Values followed by different superscripts in the same row are significantly different at α<0.05. * Comparison is made with the average value of niger seed and linseed.
